# Overexpression of miR-92a attenuates kidney ischemia–reperfusion injury and improves kidney preservation by inhibiting MEK4/JNK1-related autophagy

**DOI:** 10.1186/s11658-023-00430-3

**Published:** 2023-03-08

**Authors:** Ming Ma, Hui Li, Saifu Yin, Tao Lin, Turun Song

**Affiliations:** 1 Department of Urology, West China Hospital, Sichuan University, 37# Guoxue Alley, Chengdu, 610041 Sichuan China; 2 Organ Transplantation Center, West China Hospital, Sichuan University, 37# Guoxue Alley, Chengdu, 610041 Sichuan China

**Keywords:** miR-92a, Kidney transplant, Ischemia–reperfusion injury, Autophagy, Apoptosis

## Abstract

**Background:**

Kidney ischemia–reperfusion injury is inevitable in kidney transplantation, and is essential for primary graft dysfunction and delayed graft function. Our previous study has proved that miR-92a could ameliorate kidney ischemia–reperfusion injury, but the mechanism has not been studied.

**Methods:**

This study conducted further research on the role of miR-92a in kidney ischemia–reperfusion injury and organ preservation. In vivo, mice models of bilateral kidney ischemia (30 min), cold preservation after ischemia (cold preservation time of 6, 12, and 24 h), and ischemia–reperfusion (reperfusion time of 24, 48, and 72 h) were established. Before or after modeling, the model mice were injected with miR-92a-agomir through the caudal vein. In vitro, the hypoxia–reoxygenation of HK-2 cells was used to simulate ischemia–reperfusion injury.

**Results:**

Kidney ischemia and ischemia–reperfusion significantly damaged kidney function, decreased the expression of miR-92a, and increased apoptosis and autophagy in kidneys. miR-92a agomir tail vein injection significantly increased the expression of miR-92a in kidneys, improved kidney function, and alleviated kidney injury, and the intervention before modeling achieved a better effect than after. Moreover, miR-92a agomir significantly reduced the apoptosis and autophagy in HK-2 cells induced by hypoxia, hypoxia–reoxygenation, and rapamycin, while miR-92a antagomir had opposite effects. Furthermore, mitogen-activated protein kinase, c-Jun NH (2) terminal kinase, caspase 3, Beclin 1, and microtubule-associated protein 1 light chain 3B were inhibited by overexpression of miR-92a both in vivo and in vitro, which in turn reduced apoptosis and autophagy.

**Conclusions:**

Our results prove that overexpression of miR-92a attenuated kidney ischemia–reperfusion injury and improved kidney preservation, and intervention before ischemia–reperfusion provides better protection than after.

**Supplementary Information:**

The online version contains supplementary material available at 10.1186/s11658-023-00430-3.

## Background

The incidence and prevalence of chronic kidney disease vary globally and are reported to be around 11% in high-income countries, including the USA and Australia [1]. The higher prevalence of hypertension and diabetes in countries with a sizeable elderly population leads to a higher incidence of end-stage kidney disease (ESKD) [1]. For patients with ESKD, kidney transplantation is the optimal treatment due to higher survival and lower cardiovascular complications [2]. Currently, donation after cardiac or brain death is the primary source of organ donation worldwide. However, the great divide between the growing waiting list of kidney transplant candidates and the limited number of donor organ sources poses a great challenge for kidney transplantation. In an era of organ shortage, minimizing organ injury to safeguard the quality of donor kidneys is one of the most critical tools for organ transplant physicians to increase organ utilization and address the industry’s dilemma.

Ischemia and reperfusion injury (IRI) are inevitable in kidney transplantation, and one of the most important mechanisms for primary nonfunction or delayed graft function immediately after transplantation [3, 4]. Studies have shown that IRI can be divided into ischemia injury and reperfusion injury [5]. During renal ischemic injury, insufficient blood perfusion leads to renal ischemia and hypoxia, which leads to further structural damage and dysfunction of renal parenchymal cells. After reperfusion, amounts of oxygen radicals are generated and further impair kidney tubular epithelial cells [6]. These two processes are accompanied by a proinflammatory response, which increases renal cell apoptosis, autophagy, and even irreversible cell death [7–9]. Autophagy is the major intracellular degradation and dynamic recycling system, which produces new building blocks and energy for cellular renovation and homeostasis [10]. In renal IRI, autophagy is upregulated chiefly and is observed to have both protective and deleterious regulatory effects [11]. However, the mechanism of the switch in autophagy is unclear, and studies suggest that it may depend on the survival and death properties of Beclin 1 and its interaction with Bcl-2 family proteins [12].

MicroRNA (miRNA) is a highly conserved, small noncoding RNA molecule, which plays critical roles in various biological processes, including cell proliferation, differentiation, development, autophagy, and apoptosis [13, 14]. In addition, researchers have shown significant differences in the miRNA profiles between patients with nephropathy or transplant-related nephropathy and controls [15–17]. Studies have shown that the expression of the miR-17–92 cluster in the kidney (miR-20a, miR-17-5p, miR-18a, miR17-3p) changed significantly immediately after kidney IRI modeling and lasted for more than 1 month [18, 19]. Our previous study demonstrated that the miR-17–92 cluster ameliorates IRI-induced acute kidney injury. Interestingly, among miRNAs within the miR-17–92 cluster, miR-92a was upregulated most significantly following IRI compared with control samples [20]. Another study revealed that miR-92a-3p plays a protective role in regulating endothelial cell autophagy and cardiomyocyte metabolism [21]. Due to the dual regulation of autophagy, this study was carried out further to study the mechanism of miR-92a in renal IRI and organ cold preservation, and determine whether it is involved in regulating autophagic transition.

## Materials and methods

### Animals

Eighty-four male C57BL/6 mice (6–8 weeks old, body weight 20–22 g; Chengdu Dossy Experimental Animals, Co. Ltd., Chengdu, Sichuan Province, China) were raised following standard guidelines. The Animal Ethics Committee of West China Hospital, Sichuan University, Chengdu, China (no. 2019141A) approved this study. All experimental procedures followed the National Institutes of Health Guide for the Care and Use of Laboratory Animals.

For the first experiment, mice were randomly divided into sham, ischemia (I), ischemia and cold preservation (IC), and IRI groups. For the I group (*n* = 6), the bilateral kidney pedicles were clamped simultaneously with a noninvasive artery clamp for 30 min, and then the kidneys were removed for further experiments. For the IC group (*n* = 9), the clamps were removed after 30 min of ischemia, and then kidneys were removed and cold preserved in 4 °C UW preservation solution (6, 12, and 24 h, respectively) for the follow-up experiments. For the IRI model (*n* = 9), the clamps were removed after ischemia and reperfusion were performed for 24, 48, or 72 h, and then mice were euthanized to obtain the kidneys. The kidney pedicles were not clamped for the sham group (*n* = 6), and the other operations were the same as in the I group.

For the second experiment, Cy3-labeled miR-92a agomir or agomir NC (10 μL/g body weight, GenePharma) were injected through the tail vein of mice 24 h before modeling (*n* = 6 for each group), and all mice were sacrificed 24 h after modeling. The miR-92a agomir and agomir NC were synthesized by GenePharma Co. Ltd. (Shanghai, China). For the in vivo test, a lipofectamine–agomir complex was prepared by mixing miRNA agomir (20 nmol) with lipofectamine 2000 (200 μL) and injected into mice via the tail vein (10 μL/g body weight).

For the third experiment, the mice were subdivided into the following seven groups (*n* = 6 for each group): sham group; IC + agomir NC (IC–NC); IRI + NC (IR–NC); use agomir before ischemia in IC group (ag before IC); use agomir before ischemia in IRI group (ag before IR); use agomir before cold preservation in IC group (ag during IC); and use agomir after IRI in IRI group (ag after IR). Mice in the first five groups were given agomir or agomir NC 24 h before and sacrificed after modeling. In the ag during IC group, kidneys were removed after 30 min ischemia and preserved in 4 ℃ UW preservation solution containing agomir for 6 h. In the ag after IR group, agomir was given after ischemia. To dynamically observe the effect of agomir on renal function, serum creatinine, and urea nitrogen of mice in the sham group, IR-NC group, ag before IR, and ag after IR were measured at 48 h, 3 d, and 7 d after modeling. Blood and kidney tissues of mice were collected for the following molecular and pathological examination. The level of serum creatinine (Scr) was detected by the creatine oxidase method (Creatinine Assay Kit, Nanjing Jiancheng Bioengineering Institute, Nanjing, Jiangsu, China), and the urea nitrogen was measured by the urease method (C013-2, Nanjing Jiancheng Bioengineering Institute).

### Cell grouping and interventions

HK-2 cells were purchased from the American Type Culture Collection (ATCC, CRL-2190). HK-2 cells were cultured in serum-free DMEM/F12 medium (1552510, Gibco, Thermo Fisher Scientific Inc. Waltham, MA, USA). After synchronization, the cells were divided into three groups: control group (Con), hypoxia group (Hypo), hypoxia and reoxygenation group (Reo 6, 12, and 24 h). First, HK-2 cells were placed in an oxygen deprivation chamber (Billups-Rothenberg), and 95% N_2_ and 5% CO_2_ were mixed to induce hypoxia. The HK-2 cells were then incubated in a cell incubator at 37 ℃ for 24 h. HK-2 cells in the Reo groups were restored to normal oxygen conditions (complete culture base plus aerobic 6, 12, and 24 h) after hypoxia. To explore the effects of miR92-a on HK-2 apoptosis and autophagy under hypoxia conditions, agomir NC, miR-92a agomir (25 nM), and miR-92a antagomir (50 nM) synthesized by GenePharma Co. Ltd. (Shanghai, China), were used to intervene in the Con, Hypo, and Reo group cells, respectively.

### Histological examination

The histopathological changes in the kidneys were detected by hematoxylin–eosin (H&E) staining. The Pathology Laboratory of West China Hospital (Sichuan University, Chengdu, Sichuan Province, China) assisted us in processing pathological sections. Three slices of each mouse were statistically analyzed at ×400 magnification.

The microstructure of kidneys was observed by transmission electron microscope (TEM). The kidney slice (approximately 0.5 × 0.5 cm) was fixed in 3.0% glutaraldehyde and sent to Chengdu Li Lai Biotechnology Co. Ltd (Chengdu, Sichuan Province, China) for analysis. A JEM-1400PLUS transmission electron microscope (JEOL Ltd., Tokyo, Japan) was used for observation.

Terminal deoxynucleotidyl transferase-mediated dUTP nick end labeling (TUNEL, Roche, 11684795910) was used to detect the apoptosis of kidneys. Briefly, paraffin-embedded sections were routinely dewaxed and hydrated. Next, these slides were permeabilized by proteinase K solution. After washing with phosphate buffer solution, all slides were refixed and equilibrated with equilibration buffer for 5–10 min. Then, these slices were labeled with TdT reaction mix in a dark and moist box for 60 min. The slice was placed in 2× Saline Sodium Citrate buffer (SSC) to stop the reaction, and 4′,6-diamidino-2-phenylindole (DAPI) nuclear stain was added in the mounting medium, and analyzed. Localized green fluorescence of apoptotic cells was detected in the blue background by fluorescence microscopy (ZEN 2012 Lite, ZEISS).

### Molecular examination

The apoptosis of HK-2 cells was detected by flow cytometry. Cells in each group were digested and collected with trypsin (15050065, Gibco, Thermo Fisher Scientific Inc.) without EDTA. Then, the cells were resuspended with 100 μL binding buffer after washing with phosphate buffer solution (PBS), and 3 μL Annexin V and 3 μL PI (Annexin V-FITC staining kit, Beyotime Biotechnology Co. Ltd., Haimen, Jiangsu, China) were added then incubated for 20 min at room temperature. The cells were collected by centrifugation, resuscitated by PBS, and then detected by flow cytometry.

Total ribonucleic acid (RNA) of the kidneys was isolated using Trizol Reagent (G3013, Wuhan Servicebio Technology Co. Ltd., Wuhan, Hubei, China) and reverse transcribed to cDNA with the Revert Aid First Strand cDNA Synthesis Kit (no. K1622, Thermo Fisher Scientific Inc.). Real-time-qPCR was performed using FastStart Universal SYBR Green Master (ROX) (04913914001, Roche). All of the procedures were performed according to the kit instructions. *U6* messenger RNA (mRNA) was used to standardize the target genes, and the relative quantification of PCR products was calculated using the 2^−ΔΔCT^ method. The associated gene primer sequences are shown in Additional file 1: Table S1.

The kidneys were cut and homogenized. After the concentration of the protein isolated by centrifugation was determined (BCA, Beyotime Biotechnology Co. Ltd.), equal amounts of protein were loaded on 10.0% Tris–glycine gels (120915132, Nanjing BERKE Biology Technology Co. Ltd., Nanjing, Jiangsu, China) for electrophoresis. Proteins were wet-transferred to polyvinylidene fluoride membranes (Merck Millipore, Billerica, MA, USA) with standard procedures. The primary antibodies used were as follows: Beclin 1 (1:1000, PAJ557Hu01, Wuhan Servicebio Technology Co. Ltd.), JNK (1:1000, PAB156Hu01, Cloud-clone Co. Ltd., Wuhan, Hubei, China), LC3II/I (1:1000, 3868, Cell Signaling Technology, Danvers, MA, USA), MEK4 (1:1000, PAD564Hu01, Cloud-clone Co. Ltd.), active caspase 3 (1:2000, 9664, Cell Signaling Technology), GAPDH (1:3000, T0004, Affinity Biosciences, Cincinnati, OH, USA), p-JNK (1:1000, 4668, Cell Signaling Technology). GAPDH was used to standardize the protein expression. The images were collected by a Bio-Rad ChemiDoc MP (Bio-Rad, Berkeley, CA, USA) and then quantified by Image J software (National Institute of Health, Bethesda, MD, USA).

To detect the luciferase activity of cells in each group,the Firefly and Renilla luciferase activities of each well were detected on a full-function microplate detector. The cells were divided into five groups: (1) miR-92a-3p mimic + pGL3-control + PLR-TK; (2) miR-92a-3p mimic + pGL3-MAPK8-1798–1805 + PLR-TK; (3) miR-92a-3p mimic + pGL3-MAPK8-1798–1805-mut + PLR-TK; (4) miR-92a-3p mimic + pGL3- MAP2K4-105–112 + PLR-TK; and (5) miR-92a-3p mimic + pGL3- MAP2K4-105–112-mut + PLR-TK. According to the above experimental groups, different plasmids were transfected for 48 h. All operations were then carried out following Promega’s double luciferase report kit (E1960, Promega Corporation, Madison, WI, USA).

The autophagic flux was detected by confocal microscopy. The cells were infected with mRFP-GFP-LC3 adenovirus (HB-AP210 0001, Hanbio Biotechnology Co. Ltd., Shanghai, China) 24 h before establishing the hypoxia–reoxygenation model. After modeling, the cells were collected, fixed, sealed, and then analyzed by confocal photography. MRFP-GFP-LC3, GFP, and mRFP expressed in adenovirus containing fluorescent protein were used to label and track LC3. The decrease of GFP can indicate the fusion of lysosome and autophagosome to form autophagy–lysosome. Yellow spots represent autophagosomes and red spots represent autophagy lysosomes. The number of spots can reflect the intensity of autophagy flow.

### Statistical analysis

Continuity variables were presented as mean ± standard deviation (SD) and analyzed by GraphPad Prism 5 software (GraphPad Software, San Diego, CA, USA). A one-way ANOVA followed by a Tukey’s test, was used to compare the differences of all column pairs among multiple groups. For the difference between the two groups, a Student’s *t*-test was applied. *P* < 0.05 was considered to be significant.

## Results

### Decreased expression of miR-92a and increased autophagy are associated with mouse kidney IRI

**Fig. 1:**
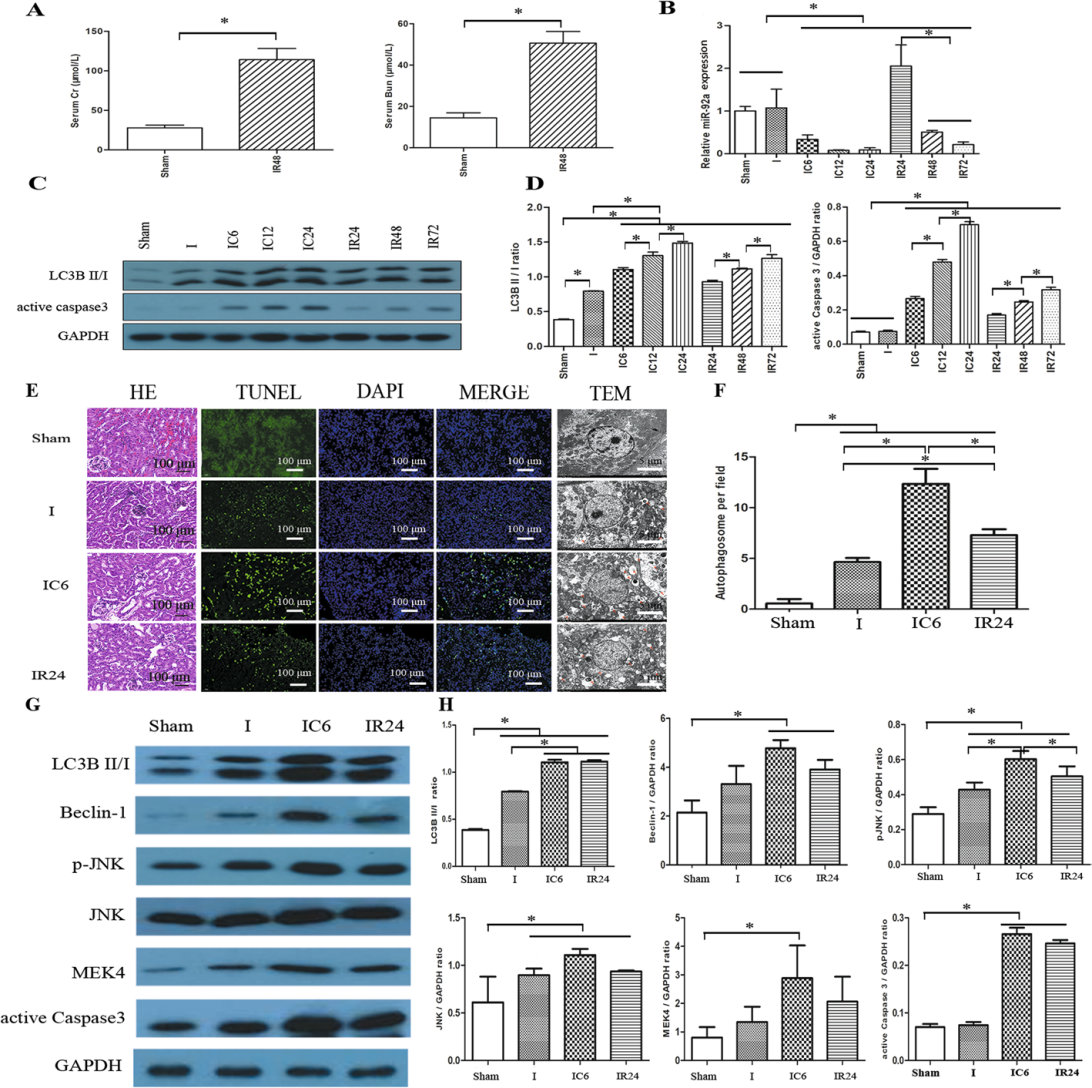
IC and IR downregulated the expression of miR-92a and increased apoptosis and autophagy in mice. **A** The content of serum Cr and BUN in the sham and IR48 groups. **B**The expression of miR-92a. Representative band **C** and statistical analysis **D** of LC3B II/I and active caspase 3 expression using Western blotting. **E** Representative images of H&E staining (×100), TUNEL (×100), and TEM (×2000) in each group. **F** The statistical results of autophagosomes in I, IC, and IR groups. Using Western blotting, the representative band **G** and statistical analysis **H** of LC3B II/I, Beclin 1, pJNK, JNK, MEK4, and active caspase 3 expression. *P < 0.05. *BUN* blood urea nitrogen, *Cr* creatinine, *GAPDH* glyceraldehyde phosphate dehydrogenase, *HE* hematoxylin–eosin, *I* ischemia, *IC* ischemia and cold preservation, *IRI* ischemia and reperfusion injury, *JNK* c-Jun NH (2) terminal kinase, *LC3B* microtubule-associated protein 1 light chain 3B, *MEK4* mitogen-activated protein kinase, *pJNK* phosphorylated JNK, *TEM* transmission electron microscope, *TUNEL* terminal deoxynucleotidyl transferase (TdT)-mediated dUTP nick end labeling

The elevated levels of SCr and blood urea nitrogen (BUN) (*P* < 0.05, Fig. 1A) suggested that the IR model was successfully established. Compared with the sham group, the expression of miR-92a was not induced by ischemia (*P* = 0.107), but the expression of miR-92a was significantly decreased with time in IC and IR groups (*P* < 0.05, Fig. 1B). Compared with the sham group, IC and IR treatment upregulated the expression of LC3B II/I and active 3 in a time-dependent manner (all *P* < 0.05, Fig. 1C, D). The IC6 and IR24 groups were relatively high among the modeling groups: therefore, to further explore the effect of miR-92a on autophagy and apoptosis, we chose these two timepoints (IC6 and IR24) for subsequent experiments. H&E staining revealed severe tissue injury in the IC6 group, worse than those from IR24 and I groups; TUNEL detected a similar apoptotic pattern among those groups; TEM showed autophagosomes in the I group, while more autophagosomes were found in the IC6 and IR24 groups (all *P* < 0.05, Fig. 1E, F). Moreover, the expression of LC3B II/I, Belin-1, pJNK, JNK, and active caspase 3 (all *P* < 0.05) were significantly upregulated in IC6 and IR24 groups; while the expression of active caspase 3 (*P* = 0.9789), Belin-1 (*P* = 0.6222), and MEK4 (*P* = 0.6419) was only numerically upregulated in the I group.

### Decreased expression of miR-92a is associated with increased autophagy in HK-2 cells.

**Fig. 2:**
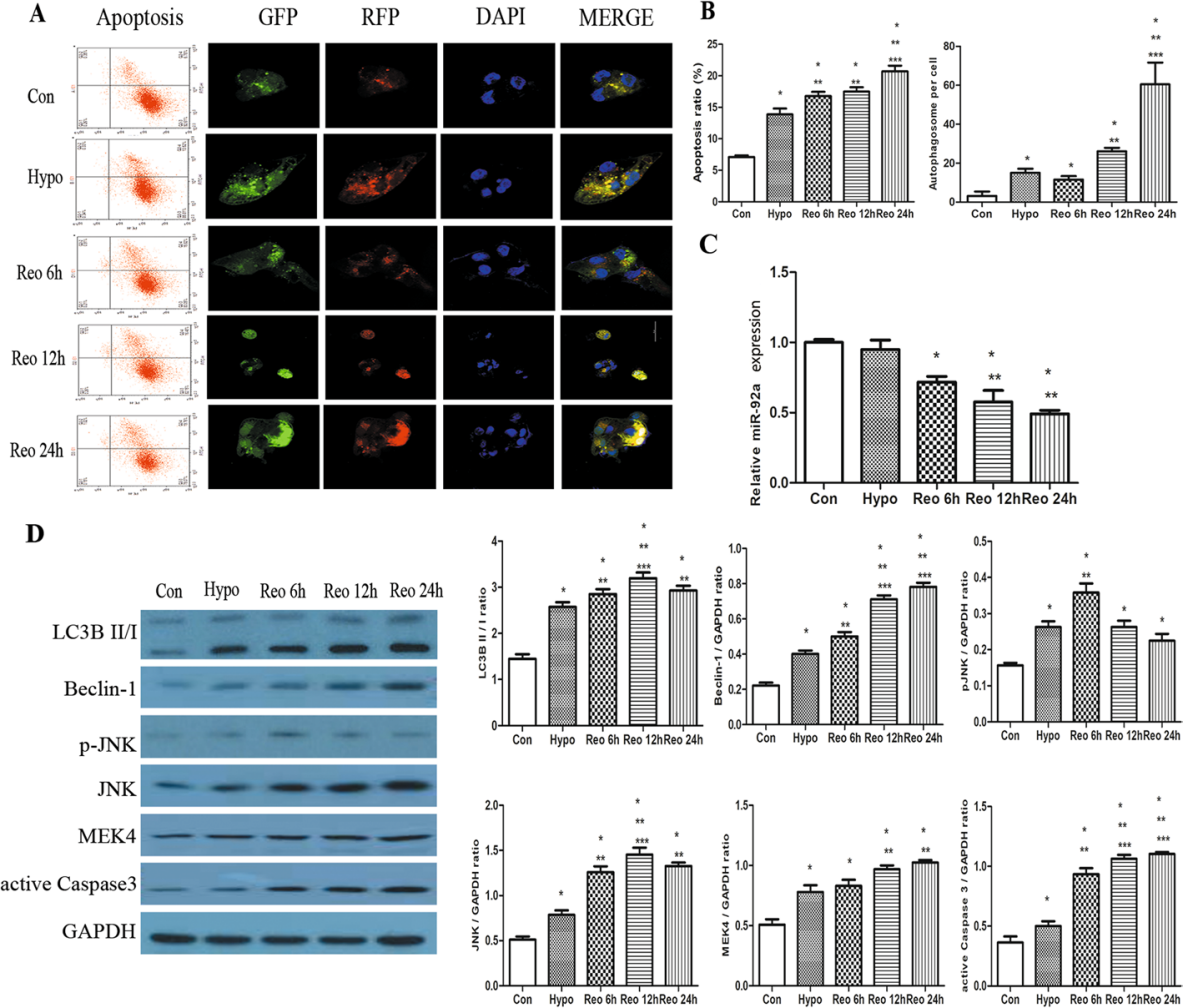
Hypoxia and reoxygenation increased apoptosis and autophagy of HK-2 cells. Representative images **A** and statistical results **B** of HK-2 cells apoptosis and autophagy among different groups (original magnification ×200). **C**The expression of miR-92a. Using Western blotting, representative band and statistical analysis **D** of LC3B II/I, Beclin 1, pJNK, JNK, MEK4, and active caspase 3 expression. *P < 0.05. *Con* control, *DAPI* 4′,6-diamidino-2-phenylindole, *GAPDH* glyceraldehyde phosphate dehydrogenase, *GFP* green fluorescent protein, *hypo* hypoxia, *JNK* c-Jun NH (2) terminal kinase, *LC3B* microtubule-associated protein 1 light chain 3B, *MEK4* mitogen-activated protein kinase, *pJNK* phosphorylated JNK, *Reo* reoxygenation, *RFP* red fluorescent protein

The experiment on HK-2 cells also found that hypoxia and reoxygenation after hypoxia could induce apoptosis, and the apoptosis rate was positively correlated with the time of reoxygenation. The autophagy flux of HK-2 cells in the Hypo group was higher than that in the Con group, while that in Reo group was the highest, and the autophagy flux increased with time (*P* < 0.05, Fig. 2A, B). The expression trend of miR-92a in HK-2 cells was similar to that in animal experiments, and the expression of miR-92a in Reo groups was significantly downregulated and decreased with time (*P* < 0.05, Fig. 2C). Compared with the control group, protein expression of HK-2 cells in the Hypo and Reo groups were significantly upregulated in a time-dependent manner (except pJNK) (all *P* < 0.05, Fig. 2D).

### Overexpression of miR-92a inhibited MEK- and JNK-mediated autophagy and apoptosis

**Fig. 3:**
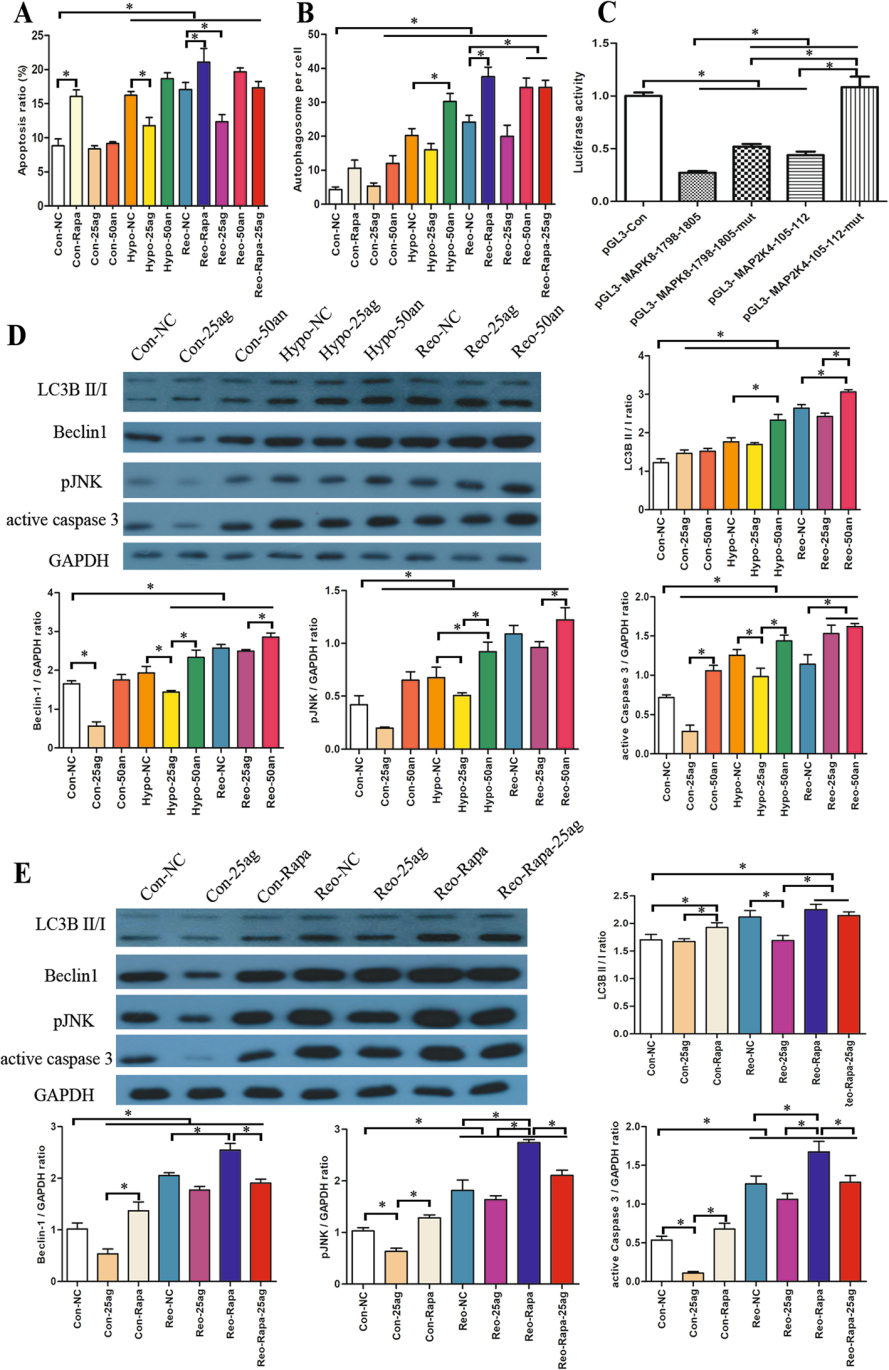
Regulation of autophagy and apoptosis in HK-2 cells by miR-92a.The apoptotic rate **A** and the number of autophagosomes **B** of HK-2 cells in each group were compared. **C** Luciferase reporter assay found that miR-92a agomir targeted 3′-UTR of MEK and JNK. Representative band and statistical analysis of regulating miR-92a on the protein expression **D** of hypoxia and reoxygenation groups and the protein expression **E** of Rapa-treated groups. *P < 0.05. *Con* control, *GAPDH* glyceraldehyde phosphate dehydrogenase, *Hypo* hypoxia, *LC3B* microtubule-associated protein 1 light chain 3B, *NC* negative control, *pJNK* phosphorylated c-Jun NH (2) terminal kinase, *Rapa* rapamycin, *Reo* reoxygenation, *25ag* 25 nM miR-92a agomir, *50an* 50 nM miR-92a antagomir

miR-92a agomir ameliorated the apoptosis of HK-2 cells induced by hypoxia and reoxygenation (Additional file 2: Fig. S1). Rapamycin (Rapa), an autophagy enhancer, increased the apoptosis rate of HK-2 cells, while miR-92a agomir partially reversed the increased apoptosis and the miR-92a antagomir cancelled this reversal (*P* < 0.05, Fig. 3A). In addition, the miR-92a agomir had a similar decrease in autophagy, while the miR-92a antagomir partially upregulated the autophagy (*P* < 0.05, Fig. 3B). Luciferase reporter assay found that the miR-92a agomir targeted 3′-UTR of MEK and JNK (*P* < 0.05, Fig. 3C). The miR-92a antagomir and Rapa further upregulated the protein expression of LC3B II/I, Beclin 1, pJNK, and active caspase 3 in HK-2 cells treated with Hypo and Reo, while miR-92a agomir partially reversed the overexpression of these proteins (all *P* < 0.05, Fig. 3D, E).

### Overexpression of miR-92a alleviated renal injury in IC and IR mice

**Fig. 4:**
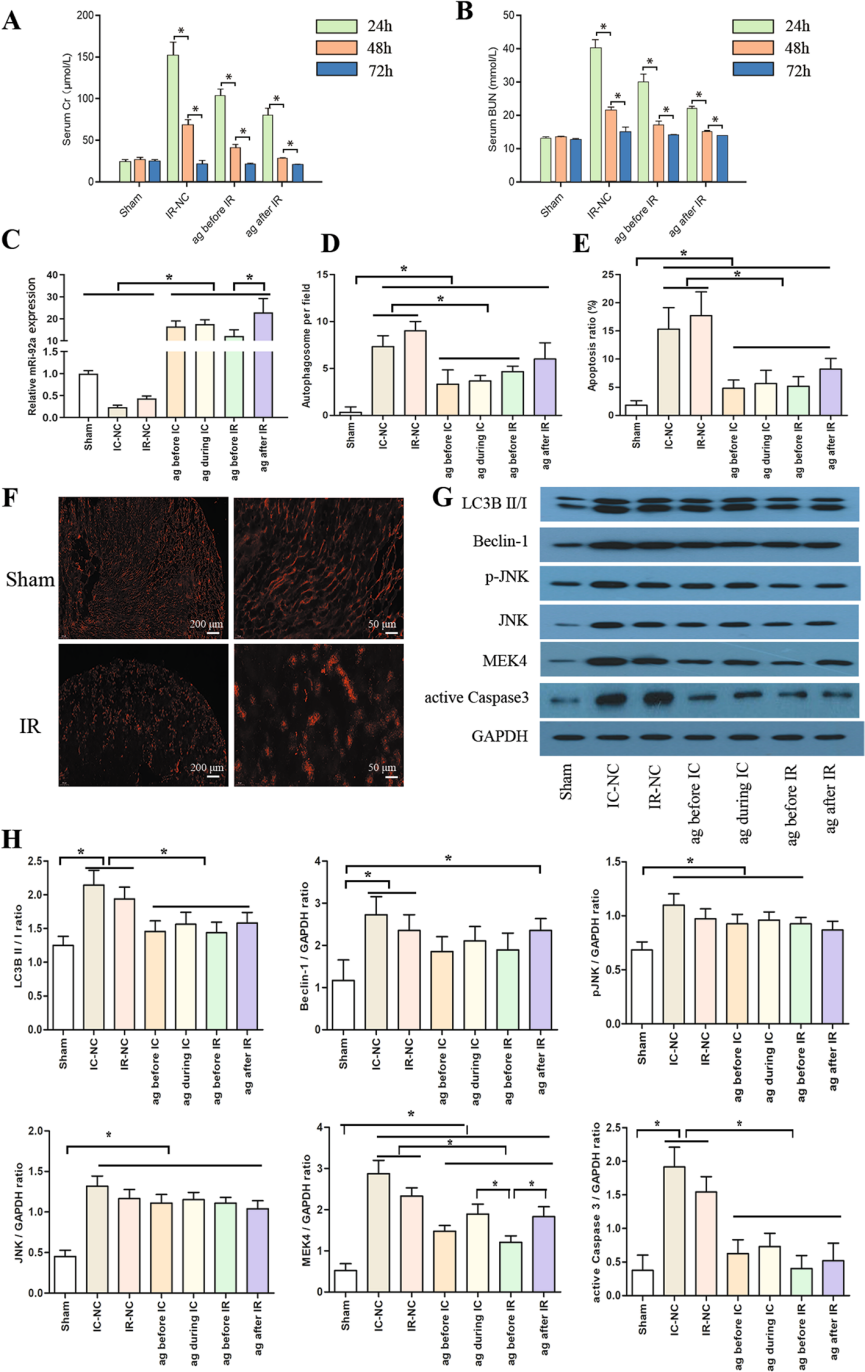
Effect of miR-92a on IC and IR kidneys in mice. The content of serum Cr **A** and BUN **B** among groups. The expression of miR-92a **C** among groups. Each group’s number of autophagosomes **D** and kidney apoptotic rate **E** were compared. The kidneys homogeneously absorbed the Cy-3 labeled miR-92a-agomir injected via tail vein (**F**). In IC and IR mice treated with miR-92a agomir, the representative band **G** and statistical analysis **H** of LC3B II/I, Beclin 1, pJNK, JNK, MEK4, and active caspase 3 expression using Western blotting. *P < 0.05. *ag* 25 nM miR-92a agomir, *BUN* blood urea nitrogen, *Cr* creatinine, *GAPDH* glyceraldehyde phosphate dehydrogenase, *IC* ischemia and cold preservation, *IR* ischemia and reperfusion injury, *JNK* c-Jun NH [2] terminal kinase, *LC3B* microtubule-associated protein 1 light chain 3B, *MEK4* mitogen-activated protein kinase, *NC* negative control, *pJNK* phosphorylated JNK

miR-92a agomir improved the kidney function of the IRI mice model (*P* < 0.05, Fig. 4A, B). After administration of miR-92a agomir, the expression of miR-92a in the kidney of mice in the IC and IR groups were significantly upregulated (*P* < 0.05, Fig. 4C). Furthermore, miR-92a agomir significantly reduced the tissue injury, autophagosome, and apoptosis of kidneys in IC and IR groups, and more remarkable improvement was detected in those receiving miR-92a agomir before the ischemia of the kidney (*P* < 0.05, Fig. 4D, E) and Additional file 2: Fig. S2). Cy-3 labeled miR-92a agomir given via tail vein injection revealed homogeneous uptake of miR-92a agomir by the kidney (Fig. 4F). In IC and IR mice treated with miR-92a agomir, the expressions of LC3BII / I, MEK4, and active caspase 3 were significantly decreased (all *P* < 0.05, Fig. 4G, H), while the expression of p-JNK (*P* = 0.0836), JNK (*P* = 0.0836), and Beclin 1 (*P* = 0.0939) were numerically decreased.

## Discussion

There are three commonly used models of kidney IRI: bilateral kidney pedicle clamping [22]; unilateral kidney pedicle clamping, with one side left as control [23]; and unilateral kidney pedicle clamping and contralateral resection as normal control [24]. According to the previous research and our experience, we chose bilateral kidney pedicle clamping for 30 min and reperfusion for 48 h to establish an IRI model. Decreased kidney function and histopathological injuries indicated that ischemia and an IRI model were successfully established. Furthermore, because the kidney would experience cold ischemia for a while during kidney transplantation, we also designed an IC model to simulate the process of cold kidney ischemia. We found that cold ischemia significantly aggravated tissue damage after warm ischemia, which was even more severe than that caused by IRI. This effect may be because the tissue is in a state of anaerobic glycolysis during cold preservation when the content of adenosine triphosphate (ATP) decreases and the production of oxygen free radicals increases, which leads to apoptosis and injury of kidney tubular epithelial cells [25]. These results suggest that it is necessary to reduce cold ischemia time or improve the quality of organ preservation under cold ischemia.

Autophagy induced during kidney IRI also plays a vital role [11]. Our previous studies also showed that the miR-17–92 miRNA expression in the kidney of the IRI model increased significantly, of which miR-92a increased most significantly[20]. This study found that the expression of LC3B II/I and caspase 3 increased with time, while the expression of miR-92a decreased with time. The expression of miR-92a in IC6 and IR24 groups is relatively the highest.To further explore the effect of miR-92a on autophagy and apoptosis, we chose these two timepoints for subsequent experiments. Regulation of several shared proteins in the autophagy and apoptosis pathways determines the cell fate of survival or death [5]. Caspase-mediated degradation of several autophagy-regulating proteins restricts autophagosome formation and thus limits autophagy [26, 27]. The apoptosis inhibitors Bcl-2 and Bcl-XL also inhibit autophagy by binding to Beclin 1 [12]. In this study, the expression trend of apoptosis-related protein active caspase 3 was consistent with kidney apoptosis, suggesting that overexpression of miR-92a might inhibit apoptosis. TEM scan showed that autophagosomes increased significantly in IC and IR groups, in a time-dependent manner. Similar results were found in the autophagy flow of HK-2 cells detected by confocal microscopy. The autophagy-associated protein LC3B II/I also increased significantly, suggesting autophagy was activated. This result is consistent with previous studies [28, 29]. The enhancement of autophagy was consistent with the downregulation of miR-92a, suggesting that miR-92a might be involved in mediating autophagy in the process of IRI. Interestingly, administration of miR-92a agomir before modeling IR can protect kidney function and reduce kidney apoptosis and autophagy better than after. The ability of preconditioning to limit renal IRI has now been demonstrated in multiple different animal models [30]. This study also showed that perfusion of UW solution containing miR-92a reduced kidney tissue injury, apoptosis and autophagy, and administration of miR-92a before modeling could better protect kidney structure and reduce apoptosis.

We further investigated the specific mechanism of miR-92a in kidney IRI-induced autophagy and apoptosis by in vitro experiment. The present study showed that miR-92a agomir significantly inhibited autophagy and reduced apoptosis in the Hypo and Reo groups of HK-2 cells. Rapa, a commonly used autophagy activator, further enhanced autophagy and apoptosis of HK-2 cells, but miR-92a agomir could only partially downregulate autophagy and apoptosis induced by Rapa. The incomplete regulation of autophagy and apoptosis by miR-92a may be related to other regulatory pathways. Bioinformatics analysis shows that MKK4 and JNK1 are target molecules of miR-92a. JNK1 can upregulate the expression of several apoptotic proteins and promote apoptosis [31]. Beclin 1 is one of the critical proteins in the regulation of autophagy, which is involved in the formation of the autophagy membrane [32]. Luciferase assay and Western blotting confirmed that miR-92a could inhibit the expression of MEK4 and JNK1 and further inhibit the level of autophagy. Therefore, we speculate that miR-92a can reduce IRI kidney injury by directly inhibiting MEK4/JNK1-mediated apoptosis and indirectly inhibiting the Beclin 1-mediated autophagy signal. After injection of Cy3-miR-92a agomir through the tail vein, the Cy3-miR-92a agomir could also be seen in the kidney tissue of the sham group, indicating that exogenous miR-92a agomir could reach and be widely distributed in kidneys. In contrast, in the IR group, the distribution of Cy3-miR-92a agomir in the kidney medulla decreased, but the epithelial cells of tubules in the kidney cortex showed aggregated distribution, suggesting that these injured tubules could absorb more Cy3-miR-92a agomir. Thus, exogenous Cy3-miR-92a agomir alleviated the pathological damage of kidney tissue caused by IRI and promoted the recovery of kidney function. It also reduced the number of apoptotic cells and the level of autophagy in kidney tissue. The detection of autophagy and apoptosis-related protein expression also confirmed the protective effect of exogenous overexpression of miR-92a.

There are still some limitations in this study. In this study, cell tests in vitro and animal experiments in vivo confirmed that miR-92a could reduce IRI-induced kidney injury, and its mechanism may be related to the downregulation of apoptosis and autophagy. However, we performed bidirectional validation on cellular experiments, but only investigated the effect of overexpression of miR-92a on IRI kidneys without reverse validation using miR-92a antagonists. In addition, this study only investigated the effect of miR-92a on IRI-induced renal apoptosis and autophagy, and overexpression of miR-92a could not completely reverse the damage of HK-2 cells induced by Rapa and hypoxia, nor can it completely reverse the damage of kidneys induced by IRI. These limitations indicate that further experiments are needed to explore the multiple regulatory mechanisms of autophagy and apoptosis. Finally, we only investigated the short-term effects of overexpression of miR-92a on IRI kidneys, and its long-term effects still need further validation.

## Conclusions

Overexpression of miR-92a attenuates kidney IRI and improves kidney preservation by inhibiting MEK4/JNK1-related autophagy, and intervention before IRI provides better protection than after.

## Supplementary Information

**Additional file 1: **Gene primer sequences.

**Additional file 2:** Representative images of apoptosis and autophagy in HK-2 cells, and representative images of HE staining (×100), TUNEL (× 100), and TEM (× 2000) in mice. **Figure S1.** Representative images of HK-2 cells apoptosis and autophagy among different groups (original magnification ×200). *Con* control, *DAPI* 4′,6-diamidino-2-phenylindole, *GFP* green fluorescent protein, *Hypo* hypoxia, *NC* negative control, *Rapa* rapamycin, *Reo* reoxygenation, *RFP* red fluorescent protein, *25ag* 25 nM miR-92a agomir, *50an* 50 nM miR-92a antagomir. **Figure S2.** Representative images of HE staining (×100), TUNEL (× 100), and TEM (× 2000) in each group. *ag* miR-92a agomir, *DAPI* 4′,6-diamidino-2-phenylindole, *HE* hematoxylin–eosin, *IC* ischemia and cold preservation, *IR* ischemia and reperfusion injury, *NC* negative control, *TEM* transmission electron microscope, *TUNEL* terminal deoxynucleotidyl transferase (TdT)-mediated dUTP nick end labeling.

## Data Availability

All data generated or analysed during this study are included in this published article [and its Additional files].

## Abbreviations

BUN: Blood urea nitrogen
Con: Control
Cr: Creatinine
DAPI: 4′,6-Diamidino-2-phenylindole
GAPDH: Glyceraldehyde phosphate dehydrogenase
GFP: Green fluorescent protein
HE: Hematoxylin–eosin
Hypo: Hypoxia
I: Ischemia
IC: Ischemia and cold preservation
IRI: Ischemia and reperfusion injury
JNK: C-Jun NH(2)-terminal kinase
LC3B: Microtubule-associated protein 1 light chain 3B
MEK4: Mitogen-activated protein kinase
NC: Negative control
pJNK: Phosphorylated JNK
Rapa: Rapamycin
Reo: Reoxygenation
RFP: Red fluorescent protein
TEM: Transmission electron microscope
TUNEL: Terminal deoxynucleotidyl transferase (TdT)-mediated dUTP nick end labeling
25ag: 25NM miR-92a agomir
50an: 50NM miR-92a antagomir

## References

[1] Webster AC, Nagler EV, Morton RL, Masson P (2017). Chronic kidney disease. Lancet.

[2] Tonelli M, Wiebe N, Knoll G, Bello A, Browne S, Jadhav D (2011). Systematic review: kidney transplantation compared with dialysis in clinically relevant outcomes. Am J Transplant.

[3] Zhao H, Alam A, Soo AP, George AJT, Ma D (2018). Ischemia-reperfusion injury reduces long term renal graft survival: mechanism and beyond. EBioMedicine.

[4] de Vries EE, Hoogland PE, Wind J, Snoeijs MG, van Heurn EL (2013). Transplantation of kidneys from paediatric DCD donors: a comparison with DBD donors. Nephrol Dial Transplant.

[5] Nieuwenhuijs-Moeke GJ, Pischke SE, Berger SP, Sanders JSF, Pol RA, Struys M (2020). Ischemia and reperfusion injury in kidney transplantation: relevant mechanisms in injury and repair. J Clin Med.

[6] Smith SF, Hosgood SA, Nicholson ML (2019). Ischemia-reperfusion injury in renal transplantation: 3 key signaling pathways in tubular epithelial cells. Kidney Int.

[7] Jani A, Zimmerman M, Martin J, Lu L, Turkmen K, Ravichandran K (2011). Perfusion storage reduces apoptosis in a porcine kidney model of donation after cardiac death. Transplantation.

[8] Kimura T, Takabatake Y, Takahashi A, Kaimori JY, Matsui I, Namba T (2011). Autophagy protects the proximal tubule from degeneration and acute ischemic injury. J Am Soc Nephrol.

[9] Nakagawa S, Nishihara K, Inui K, Masuda S (2012). Involvement of autophagy in the pharmacological effects of the mTOR inhibitor everolimus in acute kidney injury. Eur J Pharmacol.

[10] Mizushima N, Komatsu M (2011). Autophagy: renovation of cells and tissues. Cell.

[11] Decuypere JP, Ceulemans LJ, Agostinis P, Monbaliu D, Naesens M, Pirenne J (2015). Autophagy and the kidney: implications for ischemia-reperfusion injury and therapy. Am J Kidney Dis.

[12] Erlich S, Mizrachy L, Segev O, Lindenboim L, Zmira O, Adi-Harel S (2007). Differential interactions between Beclin 1 and Bcl-2 family members. Autophagy.

[13] Mohr AM, Mott JL (2015). Overview of microRNA biology. Semin Liver Dis.

[14] Gebert LFR, MacRae IJ (2019). Regulation of microRNA function in animals. Nat Rev Mol Cell Biol.

[15] Chen YN, Jing H, Tang SM, Liu P, Cheng Y, Fan YL (2022). Non-coding RNAs in sepsis-associated acute kidney injury. Front Physiol.

[16] Tang J, Yao DY, Yan HY, Chen X, Wang LJ, Zhan HK (2019). The role of MicroRNAs in the pathogenesis of diabetic nephropathy. Int J Endocrinol.

[17] Sorror ML, Gooley TA, Maclean KH, Hubbard J, Marcondes MA, Torok-Storb BJ (2019). Pre-transplant expressions of microRNAs, comorbidities, and post-transplant mortality. Bone Marrow Transpl.

[18] Godwin JG, Ge X, Stephan K, Jurisch A, Tullius SG, Iacomini J (2010). Identification of a microRNA signature of renal ischemia reperfusion injury. Proc Natl Acad Sci U S A.

[19] Kaucsar T, Revesz C, Godo M, Krenacs T, Albert M, Szalay CI (2013). Activation of the miR-17 family and miR-21 during murine kidney ischemia-reperfusion injury. Nucleic Acid Ther.

[20] Song T, Chen M, Rao Z, Qiu Y, Liu J, Jiang Y (2018). miR-17-92 ameliorates renal ischemia reperfusion injury. Kaohsiung J Med Sci.

[21] Rogg EM, Abplanalp WT, Bischof C, John D, Schulz MH, Krishnan J (2018). Analysis of cell type-specific effects of MicroRNA-92a provides novel insights into target regulation and mechanism of action. Circulation.

[22] Wei Q, Dong Z (2012). Mouse model of ischemic acute kidney injury: technical notes and tricks. Am J Physiol Renal Physiol.

[23] Le Clef N, Verhulst A, D'Haese PC, Vervaet BA (2016). Unilateral renal ischemia-reperfusion as a robust model for acute to chronic kidney injury in mice. PLoS ONE.

[24] Hesketh EE, Czopek A, Clay M, Borthwick G, Ferenbach D, Kluth D (2014). Renal ischaemia reperfusion injury: a mouse model of injury and regeneration. J Vis Exp.

[25] Ponticelli CE (2015). The impact of cold ischemia time on renal transplant outcome. Kidney Int.

[26] Bell BD, Leverrier S, Weist BM, Newton RH, Arechiga AF, Luhrs KA (2008). FADD and caspase-8 control the outcome of autophagic signaling in proliferating T cells. Proc Natl Acad Sci U S A.

[27] Laussmann MA, Passante E, Dussmann H, Rauen JA, Wurstle ML, Delgado ME (2011). Proteasome inhibition can induce an autophagy-dependent apical activation of caspase-8. Cell Death Differ.

[28] Li L, Wang ZV, Hill JA, Lin F (2014). New autophagy reporter mice reveal dynamics of proximal tubular autophagy. J Am Soc Nephrol.

[29] Guan XJ, Qian YY, Shen Y, Zhang LL, Du Y, Dai HL (2015). Autophagy protects renal tubular cells against ischemia/reperfusion injury in a time-dependent manner. Cell Physiol Biochem.

[30] O'Kane D, Baldwin GS, Bolton DM, Ischia JJ, Patel O (2019). Preconditioning against renal ischaemia reperfusion injury: the failure to translate to the clinic. J Nephrol.

[31] Yue JC, Lopez JM (2020). Understanding MAPK signaling pathways in apoptosis. Int J Mol Sci.

[32] Gao Q (2019). Oxidative stress and autophagy. Adv Exp Med Biol.

