# Supplementary material for: Overexpression of miR-92a attenuates kidney ischemia–reperfusion injury and improves kidney preservation by inhibiting MEK4/JNK1-related autophagy
Source: Cell Mol Biol Lett. 2023 Mar 8;28:20. doi: 10.1186/s11658-023-00430-3 (PMC9997008; doi:10.1186/s11658-023-00430-3)
Supplement: Supplementary file 1 — Additional file 1: Gene primer sequences. [file 11658_2023_430_MOESM1_ESM.pdf]

Primer	Sequence
miR-92a-3p-R	GTCGTATCCAGTGCAGGGTCCGAGGTATTCGCACTGGATACGACCAGGCC
miR-92a-3p-F	CCATATTGCACTTGTCCC
Universal R (URP)	GTGCAGGGTCCGAGGT
U6-F	TCGCTTCGGCAGCACATA
U6-R	TTTGCGTGTCATCCTTGC
