# Supplementary figures and images for: Overexpression of miR-92a attenuates kidney ischemia–reperfusion injury and improves kidney preservation by inhibiting MEK4/JNK1-related autophagy
Source: Cell Mol Biol Lett. 2023 Mar 8;28:20. doi: 10.1186/s11658-023-00430-3 (PMC9997008; doi:10.1186/s11658-023-00430-3)

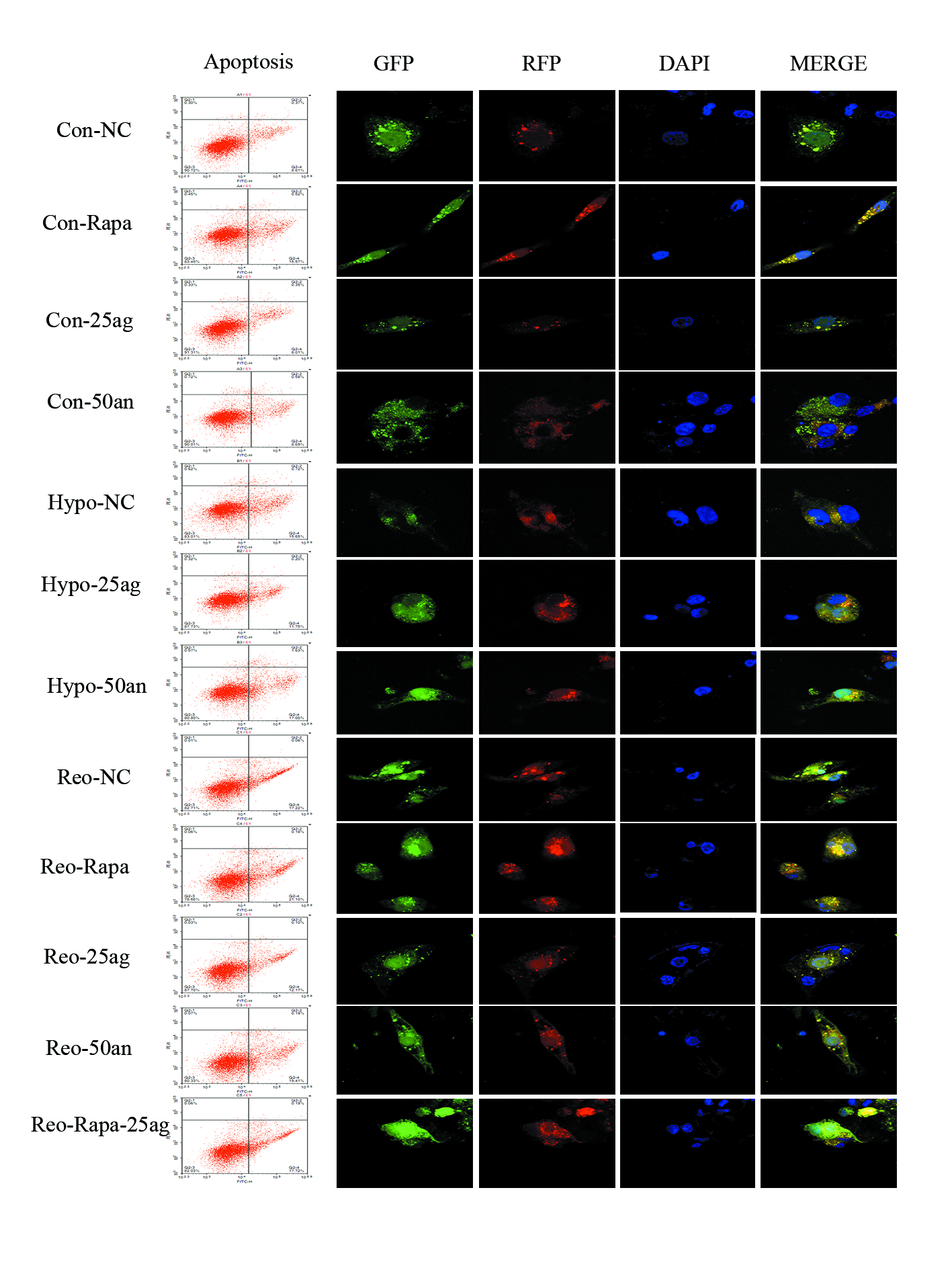

Supplement: Supplementary file 2 — Additional file 2: Representative images of apoptosis and autophagy in HK-2 cells, and representative images of HE staining (×100), TUNEL (× 100), and TEM (× 2000) in mice. Figure S1. Representative images of HK-2 cells apoptosis and autophagy among different groups (original magnification ×200). Con control, DAPI 4′,6-diamidino-2-phenylindole, GFP green fluorescent protein, Hypo hypoxia, NC negative control, Rapa rapamycin, Reo reoxygenation, RFP red fluorescent protein, 25ag 25 nM miR-92a agomir, 50an 50 nM miR-92a antagomir. Figure S2. Representative images of HE staining (×100), TUNEL (× 100), and TEM (× 2000) in each group. ag miR-92a agomir, DAPI 4′,6-diamidino-2-phenylindole, HE hematoxylin–eosin, IC ischemia and cold preservation, IR ischemia and reperfusion injury, NC negative control, TEM transmission electron microscope, TUNEL terminal deoxynucleotidyl transferase (TdT)-mediated dUTP nick end labeling. [file 11658_2023_430_MOESM2_ESM.zip › 11658_2023_430_MOESM2_ESM/Supplementary 1.tif]

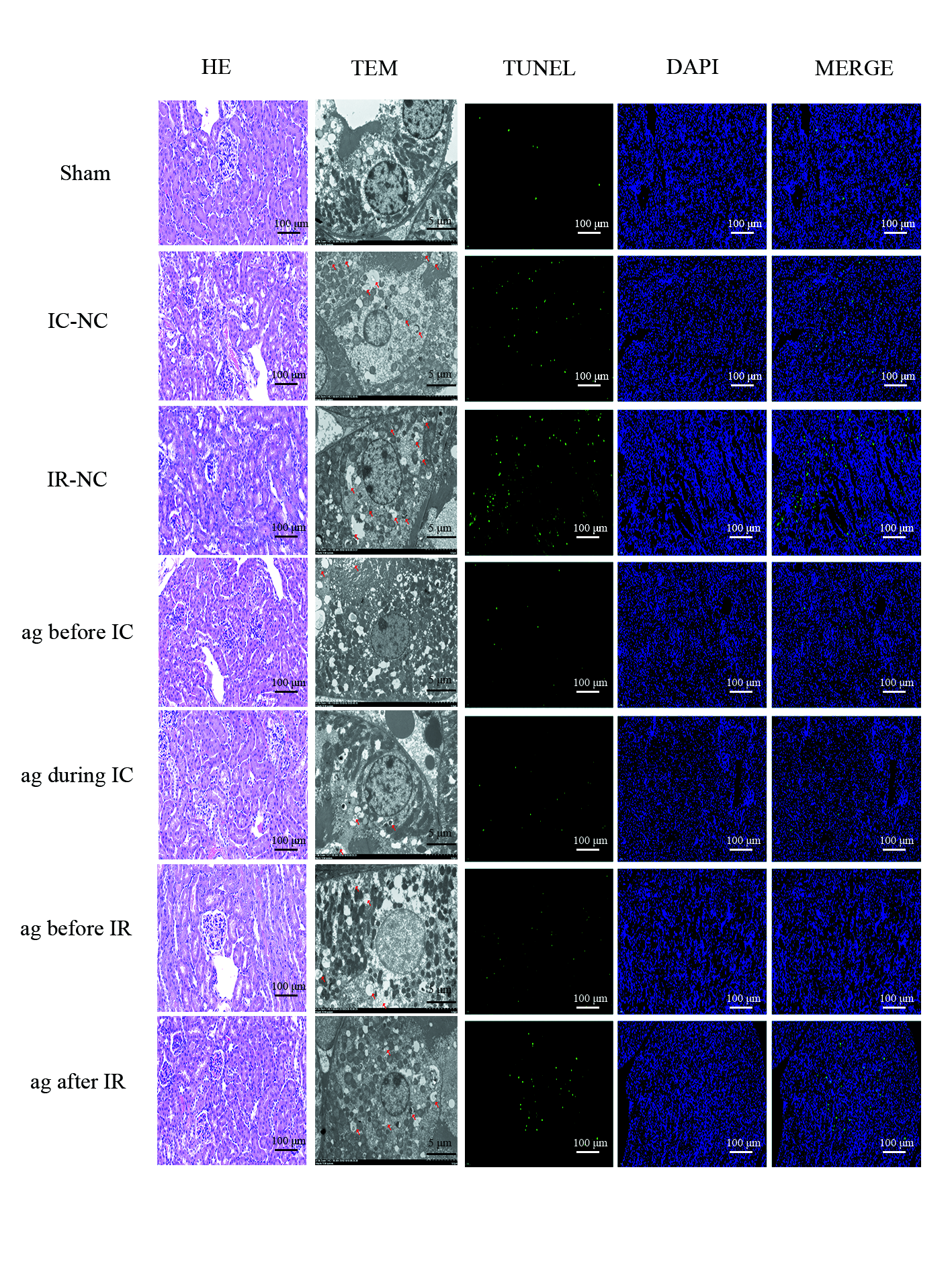

Supplement: Supplementary file 2 — Additional file 2: Representative images of apoptosis and autophagy in HK-2 cells, and representative images of HE staining (×100), TUNEL (× 100), and TEM (× 2000) in mice. Figure S1. Representative images of HK-2 cells apoptosis and autophagy among different groups (original magnification ×200). Con control, DAPI 4′,6-diamidino-2-phenylindole, GFP green fluorescent protein, Hypo hypoxia, NC negative control, Rapa rapamycin, Reo reoxygenation, RFP red fluorescent protein, 25ag 25 nM miR-92a agomir, 50an 50 nM miR-92a antagomir. Figure S2. Representative images of HE staining (×100), TUNEL (× 100), and TEM (× 2000) in each group. ag miR-92a agomir, DAPI 4′,6-diamidino-2-phenylindole, HE hematoxylin–eosin, IC ischemia and cold preservation, IR ischemia and reperfusion injury, NC negative control, TEM transmission electron microscope, TUNEL terminal deoxynucleotidyl transferase (TdT)-mediated dUTP nick end labeling. [file 11658_2023_430_MOESM2_ESM.zip › 11658_2023_430_MOESM2_ESM/Supplementary 2.tif]
